# HDR Brachytherapy in the Management of High-Risk Prostate Cancer

**DOI:** 10.1155/2012/980841

**Published:** 2012-02-22

**Authors:** Susan Masson, Raj Persad, Amit Bahl

**Affiliations:** ^1^Bristol Haematology and Oncology Centre, University Hospitals Bristol NHS Foundation Trust, Bristol BS2 8ED, UK; ^2^Department of Urology, University Hospitals Bristol NHS Foundation Trust, Bristol BS2 8HW, UK

## Abstract

High-dose-rate (HDR) brachytherapy is used with increasing frequency for the treatment of prostate cancer. It is a technique which allows delivery of large individual fractions to the prostate without exposing adjacent normal tissues to unacceptable toxicity. This approach is particularly favourable in prostate cancer where tumours are highly sensitive to dose escalation and to increases in radiotherapy fraction size, due to the unique radiobiological behaviour of prostate cancers in contrast with other malignancies. In this paper we discuss the rationale and the increasing body of clinical evidence for the use of this technique in patients with high-risk prostate cancer, where it is combined with external beam radiotherapy. We highlight practical aspects of delivering treatment and discuss toxicity and limitations, with particular reference to current practice in the United Kingdom.

## 1. Introduction

There is an ever-increasing demand for radiation techniques in the management of high-risk localised and locally advanced prostate cancer which allow dose escalation, whilst minimising the risks of acute and late severe toxicity. High-dose-rate (HDR) brachytherapy is ideally suited to achieving these goals for several reasons. 

Here we discuss the history of HDR brachytherapy for high-risk prostate cancer with relevant radiobiological principles. We summarise important existing data and relate these to current practice in the United Kingdom.

## 2. Background

Prostate cancer is the commonest malignancy in men, and in the UK approximately 40,000 cases are diagnosed annually [1]. Incidence is increasing, partly due to the increasing use of the serum PSA assay in symptomatic and asymptomatic patients. There are many patients with prostate cancer who will not die from their disease, even without treatment in some cases. However, patients with more aggressive forms of the disease require an intensive approach to treatment to maintain a normal life expectancy. 

Identifying these high-risk patients has long been a topic of debate and controversy. Improving knowledge of the biology of prostate cancer can help to guide patients in the clinic and is also of crucial importance to the design of therapeutic clinical trials: the development of increasingly aggressive treatments with the aim of cure has undoubtedly led to improved survival outcomes over time, but the risk of potentially serious short- and long-term iatrogenic side effects from “overtreating” patients at low risk should never be overlooked.

Surgical and radiotherapy series, including the use of pelvic lymphadenectomy [2, 3], have led to the identification of several factors associated with microscopic lymph node involvement and subsequent metastatic disease. These include serum PSA at presentation, Gleason score on biopsy, and clinical T (tumour) stage determined by clinical examination and, in later studies, by magnetic resonance imaging. In the UK risk is typically categorised using groupings based on those described by D'Amico et al. (Table 1) [2, 4].

In addition, an estimate of risk of lymph node involvement can be obtained using the Roach formula [5]:

(1) $$\begin{array}{cc} & \text{\%}\;\text{risk}\;\text{microscopic}\;\text{lymph}\;\text{node}\;\text{involvement}\\ & \;\;=\left(\frac{2}{3}\;\text{PSA}\right)+\left[\left(\text{Gleason}\;\text{score}\;-\;6\right)\times 10\right]. \end{array}$$

## 3. Radiotherapy in High-Risk Prostate Cancer

Favourable long-term local control rates and overall survival are seen when high-risk patients are treated with primary radiotherapy in combination with the addition of androgen suppression, prior to and after radiotherapy [6–9]. This approach has therefore become a standard of care for patients with high-risk disease. The duration of long-term adjuvant androgen suppression after radiotherapy varies in published studies, but improvements in progression-free survival have been demonstrated where 2 or 3 years of androgen suppression are used, compared with durations of 6 months or less [8, 9].

Furthermore, although no direct comparison with radiotherapy has been made, surgical series have demonstrated that following radical prostatectomy a proportion of high-risk patients will later relapse following surgery, particularly where surgical margin positivity and/or extraprostatic disease are present [10–12]. Some of these patients may be cured with radiotherapy to the prostate bed at relapse, but at the expense of exposure to potential serious long-term toxicities from both surgery and external beam radiotherapy (EBRT). Prostatectomy for patients with high-risk disease is thus considered only in carefully selected patients [13].

The optimal radiotherapy treatment volumes for patients with high-risk disease remain unclear. There are data that support the practice of moderate-dose whole pelvic radiotherapy (WPRT) in this setting [14, 15], with a high-dose “boost” to the prostate itself. The relative contributions of WPRT and long-term androgen suppression are difficult to separate, however [15], and this approach is not without acute and late toxicity, particularly gastrointestinal complications. Studies to further evaluate the role of WPRT in high-risk patients are ongoing.

## 4. Background and Rationale for HDR Brachytherapy

### 4.1. Dose Escalation

*In vitro *and clinical studies show that there is correlation between prostate cancer survival endpoints and increasing radiation dose [16–18]. However, in practice this may be at the expense of increased toxicity, due to exposure of organs at risk where conventional conformal megavoltage photon external beam radiotherapy (EBRT) is employed.

For example, a 2002 randomised study of 305 patients treated at the MD Anderson Cancer Centre [18] demonstrated a reduction in clinical and biochemical failure rate at 6-year followup (64% versus 70%, *P* = 0.003) in patients who had received 78 Gy compared with those who had received 70 Gy external beam therapy to the prostate, across a range of risk groups. However, it was noted that where more than 25% of the rectum received a dose of 70 Gy or greater, the incidence of late rectal toxicity of grade 2 or greater increased to 30%. It is frequently not possible to limit the rectal dose using conventional EBRT techniques alone. Similar outcomes are seen in a number of other large phase III dose escalation studies using EBRT alone [16, 17].

### 4.2. Radiobiological Principles

Radiobiological research demonstrates that the probabilities of acute and late radiotherapy reactions vary between body tissues and tumours and between different radiotherapy dose-fractionation schedules. In particular, the likelihood of a *late *radiotherapy reaction or response is more dependent than *acute *reactions on the fraction size (dose per fraction) for a given total dose of radiation [19].

The *α*/*β* *ratio*, a means of expressing the sensitivity of a particular tissue to altered fraction size, is used to estimate the impact of a given schedule on tumour control and toxicity and enables comparisons to be made between schedules. Tissues and tumours with a low *α*/*β* ratio have a higher relative sensitivity to changes in fraction size than those with a high *α*/*β* ratio.

Fast-growing tumours have been demonstrated to have high *α*/*β* ratio (i.e., tumour responses are less dependent on fraction size; they may be more dependent on overall treatment time), whereas increasing evidence exists to support a *low α*/*β* ratio for prostate cancer, which may be as low as 1.5 Gy, in which case a hypofractionated approach (large doses in a small number of fractions) is favoured for optimal tumour control [20–23].

The EQD2 formula is frequently used to estimate the equivalent dose at 2 Gy per fraction for a given schedule:

(2) $$\text{EQD}2=D\times \frac{\left(d+\alpha /\beta \right)}{\left(2+\alpha /\beta \right)},$$

where *D* is the total dose, *d* dose per fraction, and the *α*/*β* ratio for a given tissue is used.

HDR brachytherapy lends itself to the delivery of a large radiation dose to the prostate in a small number of fractions (hypofractionation). For practical reasons (primarily the requirement to limit the number of invasive procedures and duration of patient immobility) it has in fact been necessary to deliver treatment in this way.

For example, a typical radical EBRT schedule for prostate cancer is given as 74 Gy, using a fraction size of 2 Gy.  Table 2 illustrates the EQD2 estimations for the prostate for a variety of schedules used in published HDR brachytherapy series, assuming an *α*/*β* ratio of 1.5 Gy (see Table 2).

These doses are not achievable using EBRT alone, even with the use of more modern intensity-modulated techniques. 

### 4.3. Procedures

At our centre HDR brachytherapy is delivered using the following technique.

Under sterile conditions and with regional anaesthesia and following urethral catheterisation, up to 20 blind-ended needles are inserted into and adjacent to the prostate. To guide needle insertion, a transrectal ultrasound (TRUS) probe with stepping unit and template are used. This technique is similar to that originally described for the insertion of low-dose rate ^125^I seeds [24].

2 mm axial CT images of the pelvis are taken with the implant *in situ*. The prostate, rectum, and urethra are outlined using specialist planning software, and a planning target volume is created by adding a 3 mm margin around the prostate in all dimensions.

Source dwell positions and times are determined using specialist planning software, in order to deliver 15 Gy to the PTV in a single fraction, whilst doses to organs at risk are limited according to published guidelines [25]. Treatment is delivered using a single ^192^Ir source via an afterloading unit. Following treatment the needles are removed, and once haemostasis is achieved the urinary catheter is also removed.

There is no permanent implant with this technique; thus, no long-term radiation protection issues exist. A temporary implant also allows precise dosimetric calculations, so that the technique be used in combination with EBRT. This combination strategy is of particular relevance to high-risk patients, where it may be necessary to extend the treatment volume to include areas at high risk of microscopic spread (seminal vesicles and/or pelvic lymph nodes) to a moderate dose, whilst administering a high-dose boost to the prostate. It is not possible to treat these extended “prophylactic” volumes with brachytherapy alone. 

### 4.4. Clinical Data

Although previously some centres had reported the use of iridium wire implants, the modern HDR brachytherapy prostate “boost” was first developed in the late 1980s in combination with external beam radiotherapy (EBRT) to the whole pelvis in intermediate- and high-risk patients. In an early study by the Michigan group [26], three fractions of HDR brachytherapy were given concurrently with EBRT in weeks 1, 2, and 3 of treatment. Brachytherapy was well tolerated, and 9 of the first 10 patients who underwent planned rebiopsy at 18 months after treatment were found to have no residual cancer.

With longer-term followup and comparison with a matched cohort [27], data emerged from this group to suggest superiority of HDR brachytherapy in this context, at least in terms of biochemical control, due to the much increased biologically effective dose delivered using the brachytherapy technique. An interim analysis confirmed improved biochemical control rates with two versus three fractions and with increased dose per fraction [28].

Data from this group were combined for analysis with those from two other institutions to establish the largest published series of over 600 patients [29]. A 73% 10-year biochemical control rate was observed across all treated groups, with disease-free survival of 49%, and encouraging results even in patients at high risk (69% biochemical control at 5 years). Although a variety of doses and schedules were employed between centres, encouragingly results were consistent.

Similarly, a Swedish group [30] reported 4-year follow-up data in 2005 on over 200 patients treated with EBRT to 50 Gy in 25 fractions with two fractions of HDR brachytherapy of 10 Gy each, in an interval midway through the EBRT. In the high-risk group (47 patients) overall 5-year biological no evidence of disease (bNED) was 61%.

Again, 10-year follow-up data from California (209 patients) [31] reported 69% bNED for the high-risk group.

The only prospective randomised controlled trial of EBRT alone versus EBRT with HDR brachytherapy boost, in 220 patients, reported significantly improved biochemical relapse-free survival in the brachytherapy group (5.1 years versus 4.3 years at median 30-month followup) with a reduction in acute rectal toxicity [32]. Although the dose-fractionation schedule in the EBRT arm of this study may now be considered suboptimal, these results do support previous data and the principles discussed and have supported further work evaluating dose escalation with HDR brachytherapy monotherapy in lower-risk patients [33]. It is recognised, however, that a direct comparison of HDR + EBRT and dose-escalated EBRT in intermediate- and high-risk patients has not been carried out.

A systematic review published in 2009 [34] compared results from studies evaluating high-dose (>75 Gy) EBRT, HDR brachytherapy with EBRT and low-dose rate seed brachytherapy with EBRT. Superior results, in terms of progression-free survival and overall survival, were seen for HDR brachytherapy and EBRT when compared with other techniques. This is likely to be due to the high doses achieved—it is noted that due to dose gradients within an HDR implant, there may be regions which receive far greater doses than those prescribed. The authors of this paper acknowledge that although every attempt has been made to account for confounding factors, marked variations between methods and definitions of survival endpoints in published studies mean that these results should be interpreted with a degree of caution; nevertheless these data lend further support to the practice of HDR brachytherapy.

### 4.5. Single Fraction HDR Brachytherapy

The increasing body of evidence to support hypofractionation has led to the development of an “ultra-hypofractionated” single fraction HDR boost. This has obvious potential biological, practical, and cost-saving advantages, and any geometric uncertainty is virtually eliminated as there is no risk of interfraction variability.

The EQD2 to the prostate for a single fraction of 15 Gy, using a presumed *α*/*β* ratio of 1.5 Gy, is estimated at 70 Gy using the formula described. When combined with EBRT, an equivalent dose of up to 120 y in 2 Gy fractions is achievable.

The use of single fraction HDR brachytherapy in combination with EBRT in intermediate-risk patients has been reported [35]. At relatively short followup (median 1.14 years) biochemical control rates were excellent and observed toxicity acceptable; there was a notable lack of acute and late gastrointestinal toxicity. Genitourinary toxicity is far more common, partly due to difficulty avoiding high doses to the prostatic urethra; however, no severe late genitourinary toxicity has yet been observed in this cohort of 125 patients.

There has been much interest in this technique, which has also been adopted in a number of centres for the management of high-risk patients in combination with pelvic EBRT. Further work and followup are required, however, to evaluate the true long-term consequences of ultra-hypofractionation, and caution should be exercised particularly in patients with preexisting urinary symptoms (see Section 4.6).

### 4.6. Toxicity

Acutely, HDR brachytherapy commonly leads to an increase in urinary symptoms as measured by the IPSS score [32, 35–38]. However, this is generally short lived, and catheterisation is a rare event. Relatively high rates of late grade 3 urinary toxicity have been noted in patients who undergo postradiotherapy transurethral resection (TUR), and in those with large preexisting defects from previous TUR [31] HDR brachytherapy is relatively contraindicated, therefore, in patients with significant obstructive lower urinary tract symptoms prior to treatment [25]. Incontinence is an infrequent event.

Deterioration in potency is reported after HDR brachytherapy and EBRT in high-risk patients. However, there is much variation in method of assessment and the impact of androgen deprivation therapy is difficult to separate from the effects of radiotherapy. The rate of erectile dysfunction in this patient group is however high and increases with time (up to 76% at 7 years has been reported) [25].

There is no doubt that acute and late rectal toxicty rates are low following HDR brachytherapy. In the randomised trial [32], a significant reduction in acute rectal discharge was seen in the brachytherapy group, and in the single fraction study [35] only 6.5% of patients experienced acute grade 2 or greater gastrointestinal toxicity, with 10% grade 2 late toxicity. No severe late toxicity has been reported in this group, but again the short reported follow-up period is noted.

### 4.7. HDR Brachytherapy in UK Practice

HDR brachytherapy is an attractive treatment option for patients with high-risk disease, with the potential to increase dose and thus improve tumour control, as well as reducing toxicity and, from a practical viewpoint, reducing overall treatment time. It is becoming increasingly available and is currently practised in a number of UK centres. Its use is supported by the National Institute for Health and Clinical Excellence (NICE) [4] for use in combination with external beam radiotherapy in appropriately selected intermediate- and high-risk patients with nonmetastatic prostate cancer [4, 25].

The potential advantages of HDR brachytherapy over EBRT alone should be discussed with appropriate patients. It is important, however, that clinicians and patients alike are aware of the limitations of current data, when making decisions on the optimum treatment approach.

## Figures and Tables

**Table 1:** Prostate cancer risk groups.

Group	Criteria
Low risk	T1-T2a and PSA ≤ 10 ng/mL and Gleason score ≤ 6
Intermediate risk	T2b or PSA > 10 ≤ 20 ng/mL or Gleason score 7
High risk	≥T2c or PSA > 20 ng/mL or Gleason score ≥ 8

**Table 2:** Estimated equivalent doses (2 Gy per fraction) for published HDR schedules using the EQD2 formula, assuming *α*/*β* for prostate cancer of 1.5 Gy [19].

Author	Schedule	EQD2 prostate (*α*/*β* 1.5 Gy)
Galalae et al. [36]	50 Gy WPRT, 40 Gy prostate EBRT, 2 fractions HDR (9 Gy per fraction)	94 Gy
Åström et al. [30]	50 Gy in 25 fractions EBRT, 2 fractions HDR (10 Gy per fraction)	115.7 Gy
Martinez et al. [28]	46 Gy in 23 fractions EBRT, 2 fractions HDR (11.5 Gy per fraction)	131.4 Gy
Hoskin et al. [32]	55 Gy in 20 fractions EBRT, 2 fractions HDR (8.5 Gy per fraction)	115.4 Gy

## References

[1] Cancer Research UK Prostate Cancer—UK incidence statistics. http://info.cancerresearchuk.org/cancerstats/types/prostate/incidence/.

[2] D’Amico AV, Whittington R, Bruce Malkowicz S (1998). Biochemical outcome after radical prostatectomy, external beam radiation therapy, or interstitial radiation therapy for clinically localized prostate cancer. *Journal of the American Medical Association*.

[3] Partin AW, Yoo J, Carter HB (1993). The use of prostate specific antigen, clinical stage and Gleason score to predict pathological stage in men with localized prostate cancer. *Journal of Urology*.

[4] NICE guidance CG58: Prostate Cancer. http://egap.evidence.nhs.uk/CG58/.

[5] Partin AW, Yoo J, Carter HB (1993). Re: the use of prostate specific antigen, clinical stage and Gleason score to predict pathological stage in men with localized prostate cancer. *Journal of Urology*.

[6] Fowler FJ, Barry MJ, Lu-Yao G, Wesson JH, Bin L (1996). Outcomes of external-beam radiation therapy for prostate cancer: a study of Medicare beneficiaries in three surveillance, epidemiology, and end results areas. *Journal of Clinical Oncology*.

[7] Bria E, Cuppone F, Giannarelli D (2009). Does hormone treatment added to radiotherapy improve outcome in locally advanced prostate cancer? Meta-analysis of randomized trials. *Cancer*.

[8] Cuppone F, Bria E, Giannarelli D (2010). Impact of hormonal treatment duration in combination with radiotherapy for locally advanced prostate cancer: meta-analysis of randomized trials. *BMC Cancer*.

[9] Bolla M, de Reijke TM, Van Tienhoven G (2009). Duration of androgen suppression in the treatment of prostate cancer. *The New England Journal of Medicine*.

[10] Epstein JI, Carmichael MJ, Pizov G, Walsh PC (1993). Influence of capsular penetration on progression following radical prostatectomy: a study of 196 cases with long-term followup. *Journal of Urology*.

[11] Sung MT, Lin H, Koch MO, Davidson DD, Cheng L (2007). Radial distance of extraprostatic extension measured by ocular micrometer is an independent predictor of prostate-specific antigen recurrence: a new proposal for the substaging of pT3a prostate cancer. *American Journal of Surgical Pathology*.

[12] Aydin H, Tsuzuki T, Hernandez D, Walsh PC, Partin AW, Epstein JI (2004). Positive proximal (bladder neck) margin at radical prostatectomy confers greater risk of biochemical progression. *Urology*.

[13] Heidenreich A, Bellmunt J, Bolla M (2011). EAU guidelines on prostate cancer. Part 1: screening, diagnosis, and treatment of clinically localised disease. *European Urology*.

[14] Roach M, DeSilvio M, Lawton C (2003). Phase III trial comparing whole-pelvic versus prostate-only radiotherapy and neoadjuvant versus adjuvant combined androgen suppression: radiation Therapy Oncology Group 9413. *Journal of Clinical Oncology*.

[15] Lawton CA, DeSilvio M, Roach M (2007). An update of the phase III trial comparing whole pelvic to prostate only radiotherapy and neoadjuvant to adjuvant total androgen suppression: updated analysis of RTOG 94-13, with emphasis on unexpected hormone/radiation interactions. *International Journal of Radiation Oncology Biology Physics*.

[16] Peeters STH, Heemsbergen WD, Koper PCM (2006). Dose-response in radiotherapy for localized prostate cancer: results of the Dutch multicenter randomized phase III trial comparing 68 Gy of radiotherapy with 78 Gy. *Journal of Clinical Oncology*.

[17] Dearnaley DP, Sydes MR, Graham JD (2007). Escalated-dose versus standard-dose conformal radiotherapy in prostate cancer: first results from the MRC RT01 randomised controlled trial. *The Lancet Oncology*.

[18] Pollack A, Zagars GK, Starkschall G (2002). Prostate cancer radiation dose response: results of the M. D. Anderson phase III randomized trial. *International Journal of Radiation Oncology Biology Physics*.

[19] Joiner MC, Bentzen SM, Joiner MC, Van der Kogel A (2009). Fractionation: the linear-quadratic approach. *Basic Clinical Radiobiology*.

[20] Duchesne GM, Peters LJ (1999). What is the *α*/*β* ratio for prostate cancer? Rationale for hypofractionated high-dose-rate brachytherapy. *International Journal of Radiation Oncology Biology Physics*.

[21] Brenner DJ, Martinez AA, Edmundson GK, Mitchell C, Thames HD, Armour EP (2002). Direct evidence that prostate tumors show high sensitivity to fractionation (low *α*/*β* ratio), similar to late-responding normal tissue. *International Journal of Radiation Oncology Biology Physics*.

[22] Nahum AE, Movsas B, Horwitz EM, Stobbe CC, Chapman JD (2003). Incorporating clinical measurements of hypoxia into tumor local control modeling of prostate cancer: implications for the *α*/*β* ratio. *International Journal of Radiation Oncology Biology Physics*.

[23] Arcangeli G, Saracino B, Gomellini S (2010). A Prospective phase III randomized trial of hypofractionation versus conventional fractionation in patients with high-risk prostate cancer. *International Journal of Radiation Oncology Biology Physics*.

[24] Holm HH, Juul N, Pedersen JF (1983). Transperineal 125iodine seed implantation in prostatic cancer guided by transrectal ultrasonography. *Journal of Urology*.

[25] Kovács G, Pötter R, Loch T (2005). GEC/ESTRO-EAU recommendations on temporary brachytherapy using stepping sources for localised prostate cancer. *Radiotherapy and Oncology*.

[26] Stromberg J, Martinez A, Gonzalez J (1995). Ultrasound-guided high dose rate conformal brachytherapy boost in prostate cancer: treatment description and preliminary results of a phase I/II clinical trial. *International Journal of Radiation Oncology Biology Physics*.

[27] Kestin LL, Martinez AA, Stromberg JS (2000). Matched-pair analysis of conformal high-dose-rate brachytherapy boost versus external-beam radiation therapy alone for locally advanced prostate cancer. *Journal of Clinical Oncology*.

[28] Martinez AA, Gustafson G, Gonzalez J (2002). Dose escalation using conformal high-dose-rate brachytherapy improves outcome in unfavorable prostate cancer. *International Journal of Radiation Oncology Biology Physics*.

[29] Galalae RM, Martinez A, Mate T (2004). Long-term outcome by risk factors using conformal high-dose-rate brachytherapy (HDR-BT) boost with or without neoadjuvant androgen suppression for localized prostate cancer. *International Journal of Radiation Oncology Biology Physics*.

[30] Åström L, Pedersen D, Mercke C, Holmäng S, Johansson KA (2005). Long-term outcome of high dose rate brachytherapy in radiotherapy of localised prostate cancer. *Radiotherapy and Oncology*.

[31] Demanes DJ, Rodriguez RR, Schour L, Brandt D, Altieri G (2005). High-dose-rate intensity-modulated brachytherapy with external beam radiotherapy for prostate cancer: California endocurietherapy’s 10-year results. *International Journal of Radiation Oncology Biology Physics*.

[32] Hoskin PJ, Motohashi K, Bownes P, Bryant L, Ostler P (2007). High dose rate brachytherapy in combination with external beam radiotherapy in the radical treatment of prostate cancer: initial results of a randomised phase three trial. *Radiotherapy and Oncology*.

[33] Corner C, Rojas AM, Bryant L, Ostler P, Hoskin P (2008). A Phase II study of high-dose-rate afterloading brachytherapy as monotherapy for the treatment of localized prostate cancer. *International Journal of Radiation Oncology Biology Physics*.

[34] Pieters BR, de Back DZ, Koning CCE, Zwinderman AH (2009). Comparison of three radiotherapy modalities on biochemical control and overall survival for the treatment of prostate cancer: a systematic review. *Radiotherapy and Oncology*.

[35] Morton GC, Loblaw DA, Sankreacha R (2010). Single-fraction high-dose-rate brachytherapy and hypofractionated external beam radiotherapy for men with intermediate-risk prostate cancer: analysis of short- and medium-term toxicity and quality of life. *International Journal of Radiation Oncology Biology Physics*.

[36] Galalae RM, Kovács G, Schultze J (2002). Long-term outcome after elective irradiation of the pelvic lymphatics and local dose escalation using high-dose-rate brachytherapy for locally advanced prostate cancer. *International Journal of Radiation Oncology Biology Physics*.

[37] Pellizzon ACA, Fogaroli RC, Silva MLG, Castro DG, Maia MC, Lopes A (2011). High-dose-rate brachytherapy combined with hypofractionated external beam radiotherapy for men with intermediate or high risk prostate cancer: analysis of short- and medium-term urinary toxicity and biochemical control. *International Journal of Clinical and Experimental Medicine*.

[38] Hoskin P (2008). High dose rate brachytherapy for prostate cancer. *Cancer/Radiotherapie*.

