# Corrigendum to “HDR Brachytherapy in the Management of High-Risk Prostate Cancer”

**DOI:** 10.1155/2016/6345816

**Published:** 2016-11-21

**Authors:** Susan Masson, Raj Persad, Amit Bahl

**Affiliations:** ^1^Bristol Haematology and Oncology Centre, University Hospitals Bristol NHS Foundation Trust, Bristol BS2 8ED, UK; ^2^Department of Urology, University Hospitals Bristol NHS Foundation Trust, Bristol BS2 8HW, UK

In the article titled “HDR Brachytherapy in the Management of High-Risk Prostate Cancer” [1], there was an error in Table 2, which should be corrected as follows: For Hoskin et al. [32], the dose should read 35.75 Gy in 13 fractions EBRT, not 55 Gy in 20 fractions EBRT.

## Figures and Tables

**Table 2:** Estimated equivalent doses (2 Gy per fraction) for published HDR schedules using the EQD2 formula, assuming *α*/*β* for prostate cancer of 1.5 Gy [19].

Author	Schedule	EQD2 prostate (*α*/*β* 1.5 Gy)
Galalae et al. [36]	50 Gy WPRT, 40 Gy Prostate EBRT, 2 fractions HDR (9 Gy per fraction)	94 Gy
Åström et al. [30]	50 Gy in 25 fractions EBRT, 2 fractions HDR (10 Gy per fraction)	115.7 Gy
Martinez et al. [28]	46 Gy in 23 fractions EBRT, 2 fractions HDR (11.5 Gy per fraction)	131.4 Gy
Hoskin et al. [32]	35.75 Gy in 13 fractions EBRT, 2 fractions HDR (8.5 Gy per fraction)	92.0 Gy
